# Giant gallbladder cyst with acute cholecystitis: a case report

**DOI:** 10.1186/s40792-024-02021-6

**Published:** 2024-09-19

**Authors:** Takahiro Terashi, Kohjiro Shirabe, Shoichi Inokuchi, Satoshi Tsutsumi, Atsushi Sasaki, Masahiko Ikebe, Toshio Bandoh, Junpei Wada, Shogo Urabe, Tohru Utsunomiya

**Affiliations:** 1 Department of Surgery, Oita Prefectural Hospital, Bunyo 2-8-1, Oita, 870-8511 Japan; 2 Department of Pathology, Oita Prefectural Hospital, Oita, Japan

**Keywords:** Gallbladder cyst, Acute cholecystitis, Cholecystectomy

## Abstract

**Background:**

Gallbladder cysts are rare diseases with very few reported cases, and no clinical or histological definition has been established. Furthermore, cases of giant cysts outside the gallbladder wall are extremely rare. We report a rare case of giant gallbladder cyst with acute cholecystitis.

**Case presentation:**

An 85-year-old woman with appetite loss and right lower abdominal pain lasting 2 days presented to our hospital. At first, the patient’s abdominal pain was mild to moderate with no fever. Blood tests revealed a white blood cell count of 10,950/mm^3^, and the C-reactive protein (CRP) level was 14.35 mg/dl. A contrast-enhanced computed tomography (CT) scan of the abdomen revealed a grossly distended gallbladder (14.5 × 14.5 × 8.7 cm) with an incarcerated stone in the cystic duct. The patient was treated by percutaneous transhepatic gallbladder drainage (PTGBD) with 735 ml of drainage fluid. Oral contrast magnetic resonance cholangiopancreatography (MRCP) revealed that gallbladder swelling remained (14.0 × 6.5 cm) 3 days after PTGBD. We performed laparoscopic cholecystectomy 6 days after PTGBD. Because of the severe adhesion around the junction of the cystic and common bile ducts, we performed open cholecystectomy.

The resected specimen was 14 × 11 cm in size and consisted of a gallbladder (6 × 7 cm) with a stone (2.4 × 1.8 cm) in the gallbladder and a large cystic lesion (18 × 18 cm) outside the gallbladder wall. The cystic lesion had a wall thickness of 6 to 12 mm and internal septal structures and contained hemorrhagic and necrotic tissue.

Histological examination revealed that the specimens showed a mildly swollen gallbladder and a cystic lesion on the outside of the gallbladder wall, adjacent to the gallbladder wall, with wall thickening and inflammation. The cystic lesion suggested gallbladder duplication, gallbladder diverticulum or extension of the Rokitansky-Aschoff sinus (RAS). There was no malignancy. The patient’s postoperative course was uneventful, and she was discharged 5 days after the operation.

**Conclusion:**

We present a very rare case of giant gallbladder cyst with acute cholecystitis revealed by cholecystectomy.

## Background

Gallbladder cysts are rare diseases with very few reported cases, and no clinical or histological definition has been established. The cause of gallbladder cysts is thought to be that some trigger blocks the communication between the RAS and the gallbladder lumen, causing the RAS to expand within the gallbladder wall and form a cyst, but the details are not clear [1, 2]. Furthermore, cases of giant cysts outside the gallbladder wall are extremely rare.

Herein, we present a very rare case of giant gallbladder cyst with acute cholecystitis revealed by cholecystectomy after PTGBD.

### Case presentation

**Fig. 1:**
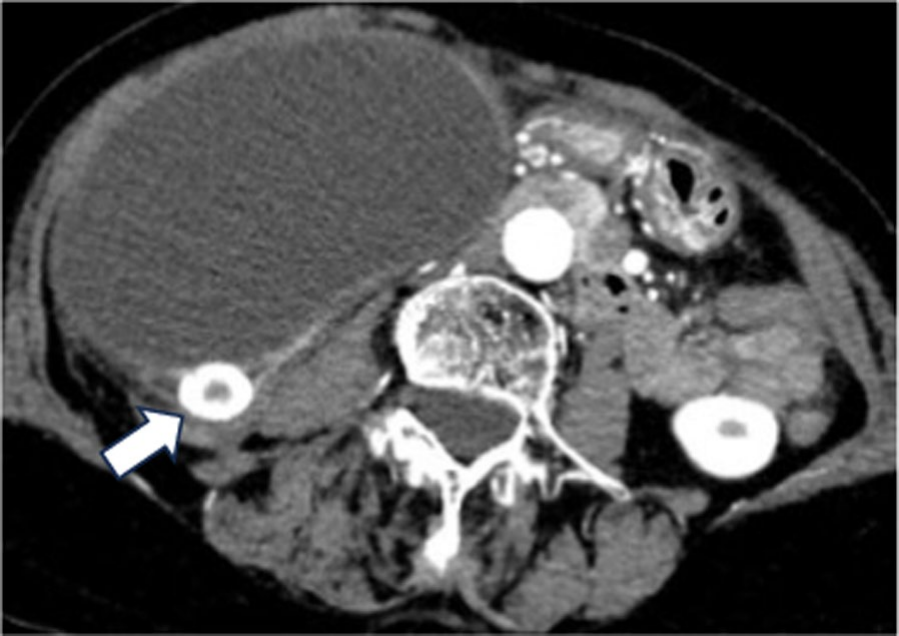
A contrast-enhanced CT scan of the abdomen revealed a grossly distended gallbladder (14.5 × 14.5 × 8.7 cm) containing a round stone (white arrow)

An 85-year-old woman with appetite loss and right lower abdominal pain lasting 2 days presented to our hospital. At first, the patient’s abdominal pain was mild to moderate with no fever. Blood tests revealed a white blood cell count of 10,950/mm^3^ and a CRP level of 14.35 mg/dl. Total bilirubin, indirect bilirubin, aspartate aminotransferase (AST), alanine transaminase (ALT), and alkaline phosphatase (ALP) were increased (3.5 mg/dl, 3.2 mg/dl, 99 U/l, 97 U/l, and 192 U/l, respectively). There was a mild increase in serum carcinoembryonic antigen (5.7 ng/ml) and a normal level of carbohydrate antigen 19–9 (21.1 ng/ml) (Table 1). Abdominal ultrasound sonography revealed a large cystic tumor in the right upper quadrant to lower quadrant with edematous wall thickness, fluid correction and a stone without a tumorous lesion, suggesting an enlarged gallbladder. A contrast-enhanced CT scan of the abdomen revealed a grossly distended gallbladder (14.5 × 14.5 × 8.7 cm) containing stone. CT scan also revealed significant gallbladder wall thickness and increased density of the surrounding adipose tissue, suggesting acute cholecystitis. In addition, impaction of a stone in the cystic duct, which was compressed to dorsal side, was suspected (Fig. 1).

**Table 1:** Laboratory data on admission

WBC 10950/μL	T.Bil 3.5 mg/dL	Amy 52 U/L
RBC 427 × 10^4^/μL	D.Bil 0.3 mg/dL	BUN 12.9 mg/dL
Hb 14.2 g/dL	I.Bil 3.2 mg/dL	Cr 0.67 mg/dL
Ht 42.8%	AST 99 U/L	CRP 14.35 mg/dL
Plt 26.8 × 10^4^/μL	ALT 97 U/L	CEA 5.7 ng/mL
TP 6.2 g/dL	LDH 181 U/L	CA19-9 21.1 U/mL
Alb 3.9 g/dL	ALP 192 U/L	
	γGTP 80 U/L

**Fig. 2:**
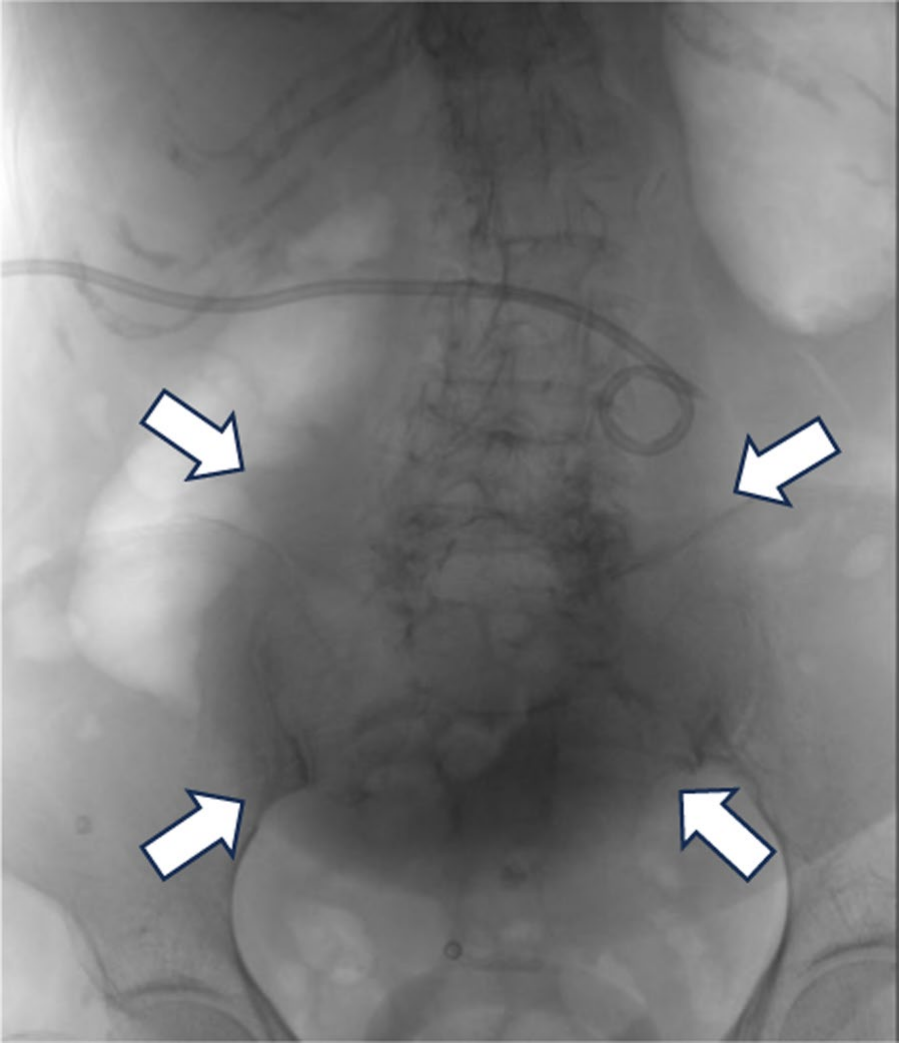
X-ray photograph showed the cavity after PTGBD insertion (white arrows)

It was difficult to perform the operation at the time because of the grossly distended gallbladder. Then, the patient was treated via PTGBD. PTGBD was performed under US guidance. A pale-yellow, transparent, and serous drainage was observed. Drainage tube was determined to be within the gallbladder, and a 10 Fr drainage tube was placed (Fig. 2). The drainage volume was 735 ml.

**Fig. 3:**
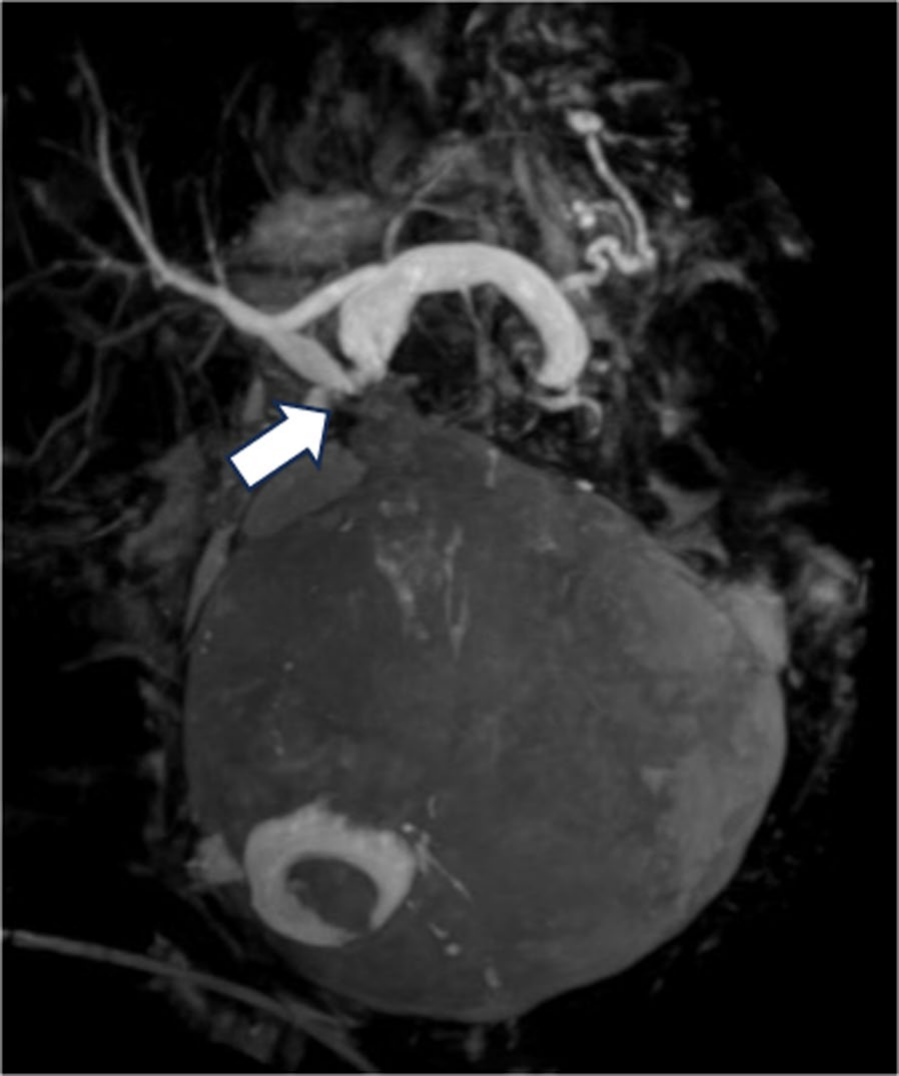
MRCP showed the V-shaped deformation of the extrahepatic bile duct with the gallbladder neck as the bending point (white arrow)

MRCP revealed that gallbladder swelling had persisted (14.0 × 6.5 cm), and a stone remained in the gallbladder neck. MRCP also showed the V-shaped deformation of the extrahepatic bile duct with the gallbladder neck as the bending point suggested gallbladder torsion (Fig. 3).

**Fig. 4:**
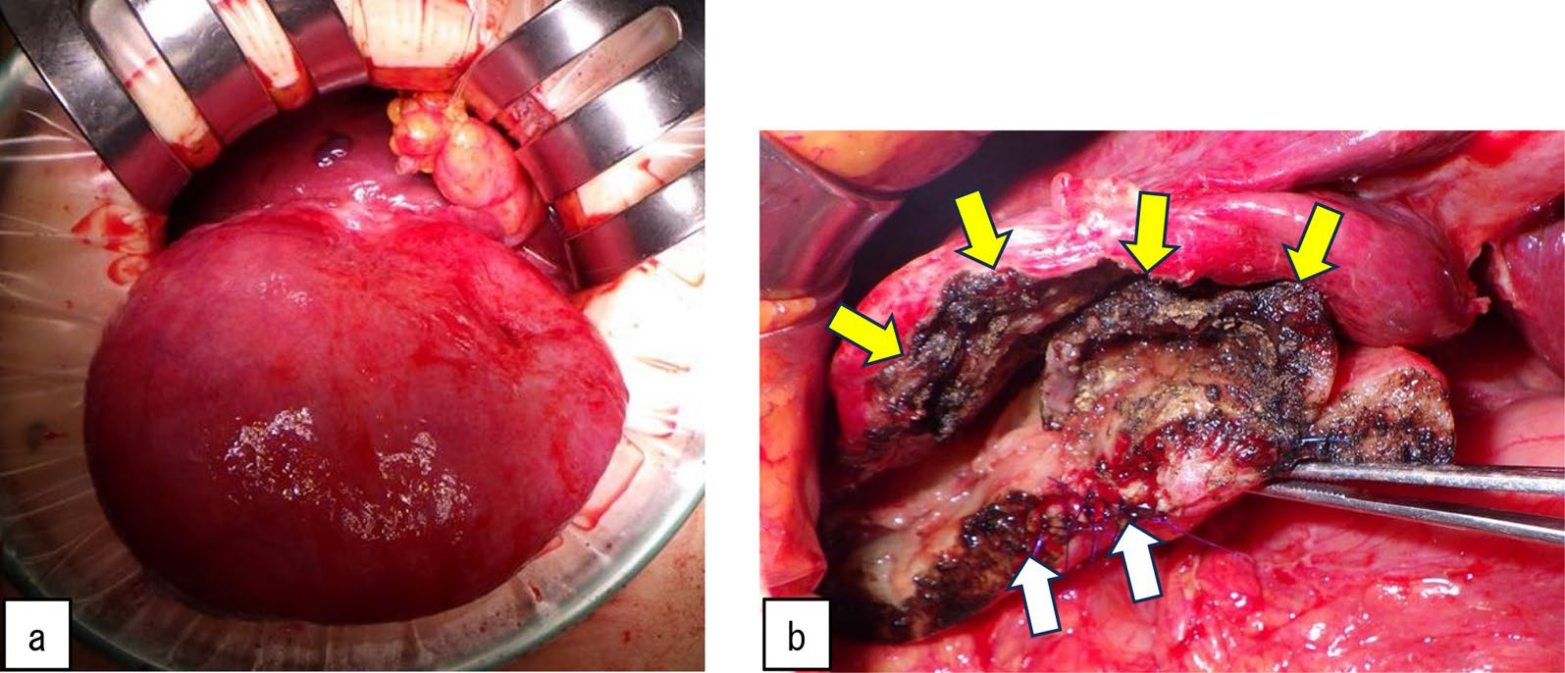
Surgical findings. Because of the severe adhesion around the junction of the cystic and common bile ducts, we performed a laparotomy (**a**). White arrows indicate a resected margin of the gallbladder and yellow arrows indicate the gallbladder bed (**b**)

We performed laparoscopic cholecystectomy 6 days after PTGBD. Because of the severe adhesion around the junction of the cystic and common bile ducts, we performed a laparotomy (Fig. 4a). After removing the adhesions, we resected the gallbladder at the neck. We found no evidence of gallbladder torsion. The lesion was not a cystic lesion protruding from the liver but a cystic lesion associated with the gallbladder that had been dissected from the gallbladder bed during surgery (Fig. 4b).

**Fig. 5:**
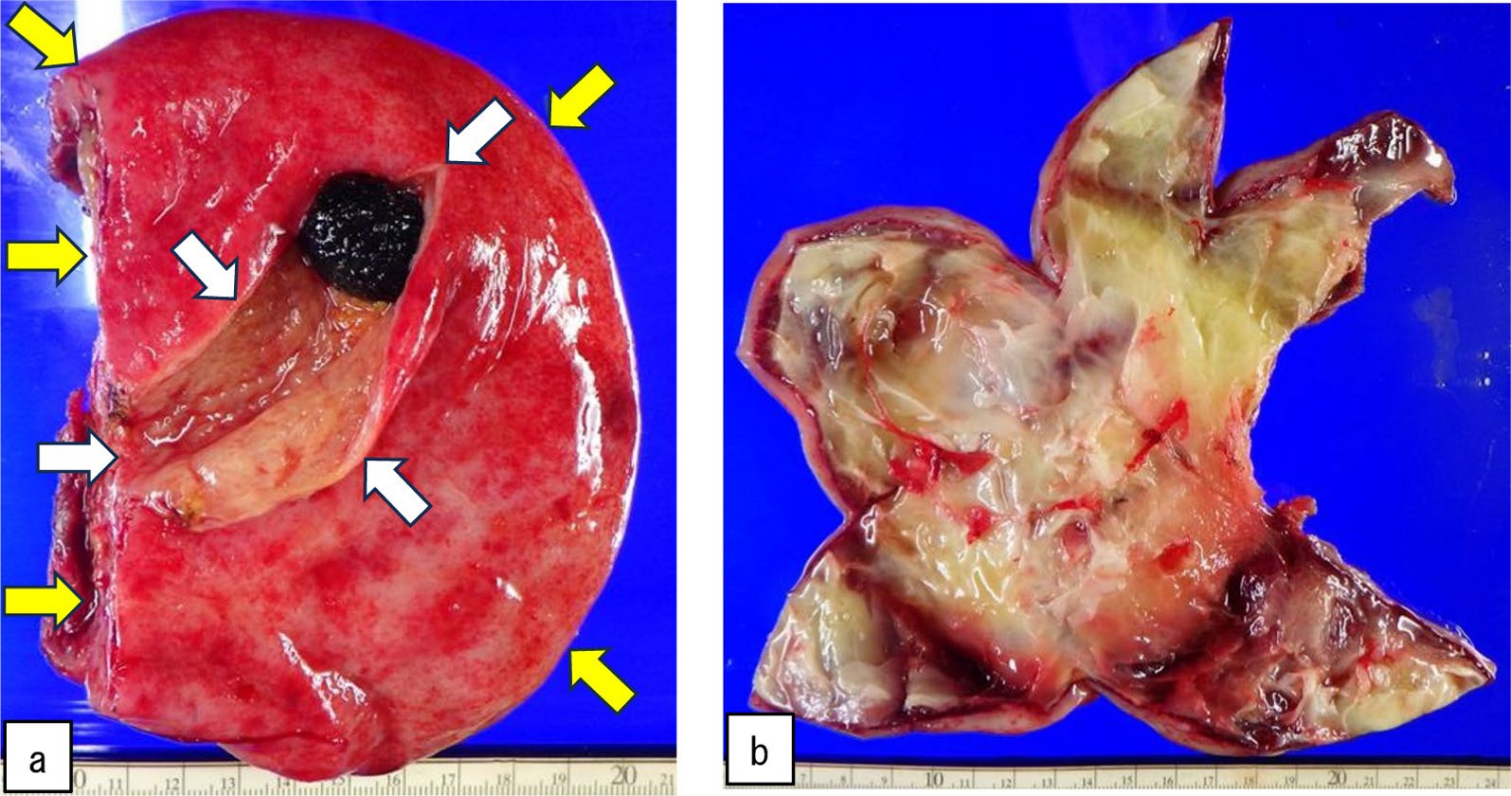
Macroscopic findings. The resected specimen was 14 × 11 cm in size and consisted of a gallbladder (6 × 7 cm) accompanied by a stone (2.4 × 1.8 cm) and around a cystic lesion (**a**, **b**). White arrows indicate the gallbladder and yellow arrows indicate the cystic lesions (**a**). The specimen shows the findings after the incision of an extramural cyst on the posterior side of the gallbladder (**b**)

The resected specimen was 14 × 11 cm in size and consisted of a gallbladder (6 × 7 cm) accompanied by a stone (2.4 × 1.8 cm) in the gallbladder and around a cystic lesion. The opened cystic lesion was 18 × 18 cm in size with gross wall thickness (6–12 mm) and contained pale yellow–reddish fluid (195 ml) accompanied by septal structures and purulent tissue inside the cavity. Macroscopically, there was no communication between the extramural cyst and the gallbladder lumen. There was no tumorous lesion (Fig. 5a, b). The patient’s postoperative course was uneventful, and she was discharged on postoperative Day 5.

**Fig. 6:**
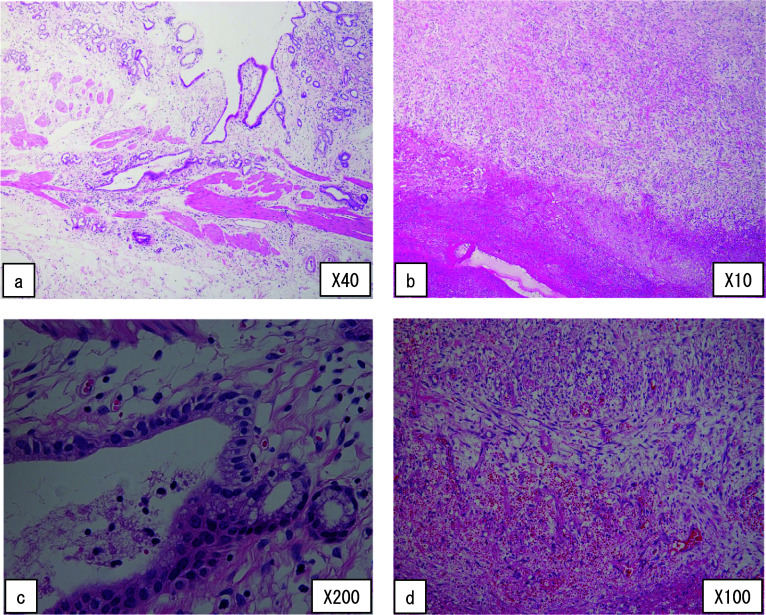
Histological findings of the resected specimen with HE staining. The specimen shows wall inflammation of the gallbladder wall (**a**) and wall thickness of the cystic lesion outside of the gallbladder wall (**b**). The specimen shows an infiltration of inflammatory cells, including neutrophils, in the gallbladder with no epithelial atypia (**c**). The specimen shows a lamellar fibrin, red blood cells in the lumen, and an infiltration of lymphocytes, neutrophils, and eosinophils in the wall of the cystic lesion. The thickened wall of the cystic lesion shows edema with proliferation of enlarged spindle cells (**d**). There was no malignancy (**a**–**d**)

Histopathological examination revealed infiltration of inflammatory cells, including neutrophils, in the gallbladder with no epithelial atypia (Fig. 6a, c), suggesting chronic cholecystitis accompanied by acute inflammation. The extramural cystic lesion showed a lamellar fibrin, and red blood cells in the lumen, and infiltration of lymphocytes, neutrophils, and eosinophils, with edematous wall thickness accompanied by proliferation of enlarged spindle cells (Fig. 6b, d). There was no malignancy (Fig. 6a–d). These findings suggested that both cholecystitis and cyst infection had occurred. A double gallbladder or a large diverticulum of the gallbladder was considered but could not be identified, because there was no epithelial component or smooth muscle layer in the wall of the cystic lesion.

## Discussion

Gallbladder cysts are rare diseases with very few reported cases, and no clinical or histological definition has been established. The first report of a gallbladder cyst was in the autopsy case of Wiedemann in 1797 in the literature by Robertson et al. [3]. Various hypotheses for the development of congenital, acquired, and neoplastic cysts have been proposed and reported in the literature. Most of these cysts originate from the RAS due to invagination of the mucosa into the wall and loss of communication with the gallbladder lumen, as observed in diverticulosis [4]. Other reports have shown the involvement of parasites, Luschka’s duct [5], aberrations of the gastric mucosa [6], and foregut cysts [7]. Cases of giant cysts outside the gallbladder wall are extremely rare. Sworn et al. reported a giant cyst at the base of the gallbladder, which was due to carcinoma in situ [8].

In this case report, abdominal contrast CT at the initial visit revealed that a stone had become impacted in the neck of the gallbladder, causing significant gallbladder enlargement and acute cholecystitis, but no gallbladder cyst was identified. Significant gallbladder enlargement remained even after PTGBD, and gallbladder torsion was suspected based on preoperative MRCP findings. Preoperatively, giant gallbladder or gallbladder torsion was suspected. At the time of PTGBD, 735 ml of fluid was drained. From the next day onwards, the drainage rate was approximately 50 ml/day, and US showed that the gallbladder enlargement was slightly improved but MRCP performed 3 days later showed that the gallbladder was still enlarged. Continued enlargement of the gallbladder was thought to be due to fluid retention caused by inadequate drainage. At the preoperative diagnosis, the gallbladder itself was deemed to be enlarged and was not considered a gallbladder cyst. Considering the findings of the resected specimen, it was thought that the cyst was punctured at the time of the PTGBD.

Kimura et al. reported a case of intramural cystic lesion of the gallbladder. In their case, preoperative contrast CT and MRI revealed a cluster of cystic lesions with a diameter of approximately 1 cm in the gallbladder lumen and body with stones, and laparoscopic cholecystectomy was performed. Histopathological findings showed a cystic lesion without epithelium lined with fibrous tissue. As findings suggesting rupture of the RAS were observed around the cystic lesion, it was speculated that the cystic lesion was formed from the RAS. The cystic epithelium was thought to have fallen off due to inflammation [9]. In our case, a large cystic lesion was formed that was continuous with the gallbladder wall, and the epithelial component was unclear. There was no obvious inflammatory cell infiltration or necrosis that penetrated the gallbladder wall or evidence of rupture of the RAS.

Giant gallbladder is a rare condition that can result from acute cholecystitis. The normal gallbladder is a pear-shaped sac that is 7 to 10 cm long and has a capacity of 30 to 50 ml [10, 11]. The gallbladder may become enlarged and distended during cystic duct or gallbladder neck obstruction due to gallstones or distal bile duct obstruction due to malignancy [12]. A giant gallbladder is usually defined as a gallbladder measuring > 14 cm in length or > 1.5 L in volume [11, 13]. Gallbladder torsion is a rare condition generally associated with a free-floating gallbladder and is observed in 8.1% of autopsies and 11.6% of surgeries in Japan [14]. A free-floating gallbladder occurs after the detachment of the gallbladder from the liver bed [15]. Intraoperative findings did not reveal any signs of gallbladder torsion in our patient. The resected specimen showed a stone in the gallbladder, but it was not impacted. In addition, the gallbladder itself was not enlarged, and a large cyst was observed outside the gallbladder wall.

The case we present here was unique, owing to the unusual structure consisting of a gallbladder and a large cyst. We assumed that a gallbladder stone became stuck in neck of the gallbladder, causing inflammation to spread to the diverticulum in the gallbladder wall or to the RAS, which expanded into a cystic shape. Histopathological examination revealed infiltration of inflammatory cells, including neutrophils, in the gallbladder, suggesting chronic cholecystitis accompanied by acute inflammation. Fibrin and red blood cells were found in the lumen of the extramural cystic lesion, and infiltration of lymphocytes, neutrophils, and eosinophils was observed. The cystic lesion and the gallbladder were continuous, but the border was unclear. The epithelial components in the lumen of the cystic lesion were unclear, and no obvious inflammatory cell infiltration or necrosis penetrating the gallbladder wall was observed. No communication between the gallbladder lumen and the extramural cyst was observed, and no malignant findings were found in either the gallbladder or the extramural cyst. Based on the histopathological findings, the differential diagnosis suggested the possibility of gallbladder duplication, gallbladder diverticulum, and cystic mass formed by inflammation spreading to the RAS dilation. However, there was no clear smooth muscle layer around the hemorrhage and inflammatory cell infiltration, no signs of transmural inflammation, and no epithelial components were observed, a definitive conclusion could not be drawn.

## Conclusions

This is a rare case of giant gallbladder cyst with acute cholecystitis revealed by cholecystectomy after PTGBD. The giant cyst was presumed to be a cystic dilation of a gallbladder diverticulum or RAS due to increased intragallbladder pressure caused by a stone lodged in the gallbladder neck. In cases of significant gallbladder enlargement, the possibility of giant gallbladder cysts should be considered.

## Data Availability

All the data generated or analyzed during this study are included in the present article.

## Abbreviations

CRP: C-reactive protein
CT: Computed tomography
PTGBD: Percutaneous transhepatic gallbladder drainage
MRCP: Magnetic resonance cholangiopancreatography
RAS: Rokitansky-Aschoff sinus
